# Mechanistic overview and suggested strategies to overcome BCL-2 inhibitor resistance in *TP53-*mutated acute myeloid leukemia

**DOI:** 10.3389/fcell.2026.1779094

**Published:** 2026-03-18

**Authors:** Umar Iqbal, Rory M. Shallis

**Affiliations:** Department of Malignant Hematology, Moffitt Cancer Center, Tampa, FL, United States

**Keywords:** acute myeloid leukemia, BCL-2 inhibitor, resistance, TP53 mutation, venetoclax

## Abstract

Venetoclax is a selective BCL-2 inhibitor that has transformed the treatment landscape for elderly and unfit patients with acute myeloid leukemia (AML). Its use has also been progressively extended to include *TP53*-mutated AML based on clinical outcomes comparable to other available standards of care. However, *TP53*-mutated AML is associated with high rates of primary and acquired resistances to venetoclax combinations, which have not afforded any meaningful gains to overall survival. The main causes for these limitations include profound genomic instability, loss of p53 pleotropic function, an immunosuppressive and exhausted marrow microenvironment, a shift away from BCL-2 dependence, defects in the post-mitochondrial executioner phase of apoptosis, lineage plasticity and monocytic differentiation, upregulation of fatty acid metabolism, and BCL-2 family gene mutations. In the present review, we discuss the pathobiology of the BCL-2 family of proteins in *TP53*-mutated AML, mechanisms of venetoclax/BCL-2 inhibitor resistance in this molecular subset, and emerging strategies to potentially overcome this deficiency to guide therapeutic management for a population of patients who are in critical need of progress.

## 1 Introduction

Identification of the B-cell lymphoma-2 gene (*BCL-2*) in the 1980s resulted in a paradigm shift in the understanding of leukemogenesis. Unlike the classic oncogenes recognized at that time (e.g., *MYC* and *RAS*) that were known to drive proliferation, the *BCL-2* gene product was found to promote cancer by inhibiting apoptosis (Tsujimoto et al., 1984; Hockenbery et al., 1990). In 2016, venetoclax became the first BCL-2 inhibitor to be approved by the United States Food and Drug Administration (USFDA). The initial approval for this drug was against relapsed/refractory (R/R) chronic lymphocytic leukemia (CLL) with 17p deletion (Stilgenbauer et al., 2016); this was later expanded to CLL without any genotypic restrictions and to acute myeloid leukemia (AML) for patients who were ineligible for intensive chemotherapy (IC) (Lasica and Anderson, 2021). The guidelines vary in their definitions of ineligibility for IC, but most include a chronological age >75 years, the Eastern Cooperative Oncology Group performance status (ECOG ≥ 3), and the presence of medical comorbidities (significant cardiopulmonary, renal, or hepatic disease) as relative contraindications to IC (Gangat and Dinardo, 2025). In 2018, the USFDA first granted accelerated approval for venetoclax usage in patients with newly diagnosed AML (who are ineligible for IC) based on two open-label non-randomized trials that reported favorable rates of complete remission (CR) and CR durations (DiNardo et al., 2019; Wei et al., 2019). Full FDA approval was subsequently obtained in 2020 based on the confirmatory and pivotal randomized phase III VIALE-A trial that reported longer median overall survival (mOS) rates (DiNardo C. D. et al., 2020). Since then, venetoclax combined with hypomethylating agents (HMAs) has become the *de facto* standard of care for the IC-ineligible population (DiNardo C. D. et al., 2020; Ferrara et al., 2013; Shimony et al., 2025).

Intensive induction chemotherapy involving cytarabine and anthracyclines has historically been the backbone of AML treatment based on its ability to rapidly debulk the disease and achieve durable remissions in younger and fit patients with chemosensitive biologies. However, the toxicity limits benefit and survival gain are modest in older and biologically adverse-risk AML patients. This has led to the adoption of venetoclax + HMA combinations owing to the improved tolerability and outpatient feasibility. Long-term follow-up data (43.2 months median follow-up) from the VIALE-A trial indicate a mOS of 14.7 months with venetoclax + azacitidine compared to 9.6 months for azacitidine alone, which has definitively established this combination as a standard of care for IC-ineligible patients with AML (Pratz et al., 2024). In real-world clinical practice, venetoclax usage has expanded well beyond the originally approved cohorts to include other myelodysplastic syndromes (MDSs) and AML subsets (Lachowiez et al., 2020; Khanam et al., 2022). Patients within specific biological subgroups, such as those classified as adverse- or intermediate-risk AML by the European LeukemiaNet (ELN) 2022 guidelines, appear to reap comparable, if not greater, benefits from the less-intensive venetoclax-based regimens than IC even when they are otherwise considered fit for IC (Shimony et al., 2025; Fathi et al., 2025). Phase II data from the randomized PARADIGM trial confirm this observation and showed 1 year event-free survival (EFS) of 53% for the venetoclax + azacitidine group compared to 39% for the IC group (Fathi et al., 2025); notably, most of the trial population was categorized as adverse risk (72%) and intermediate risk (15%) based on ELN 2022. This has provided further impetus to shift away from IC and toward venetoclax + HMA combinations for most AML subgroups irrespective of their presumed fitness for IC.

The World Health Organization (WHO, 5th edition) and International Consensus Classification (ICC) recognize *TP53-*mutated (*TP53*m) myeloid neoplasms as high-risk entities rather than treat the mutations as just prognostic modifiers (Khoury et al., 2022; Arber et al., 2022). This molecular framework recognizes the underlying biology of genomic instability and apoptotic dysfunction that drives therapeutic resistance in *TP53*m diseases. *TP53*m AML is categorized as adverse risk according to the ELN 2022 guidelines, and its mOS is approximately 6 months even in the most contemporary cohorts irrespective of patient fitness and treatment given (Sallman and Stahl, 2025). Although there is significant heterogeneity in *TP53* mutations, six hotspot missense mutations (R175H, G245S, R248Q/W, R249S, R273H/S, and R282W) within the DNA-binding domain (DBD) have been identified most commonly as the pathogenic instances (Shin, 2023). The loss of DNA binding ability secondary to DBD mutation essentially results in loss of function of TP53. A recent work suggested that the *TP53* allelic status (multihit vs. single hit) has prognostic implications in myeloid malignancies, with the *TP53* multihit status conferring a worse risk profile especially in MDS with low blasts (Bernard et al., 2020). Overall, *TP53*m AML is associated with adverse biological behaviors, including the presence of complex karyotype/genomic instabilities and resistance to currently available standard therapeutics (Daver et al., 2022). Daver et al. (2023) conducted a meta-analysis that included 12 studies and a total of 1,925 patients to quantify the effects of venetoclax + HMA vs. IC for *TP53*m AML and found no difference in the mOS (6 months); the overall response rate (ORR) of 65% vs. 41% and duration of response (DoR) of 5 months vs. 3.5 months favored venetoclax + HMA vs. IC (Daver et al., 2023; Venugopal et al., 2021). Therefore, when also considering the lesser inpatient commitment and lower rate of early mortality (3%–7% with venetoclax + HMA vs. 6%–11% with intensive induction), frontline venetoclax + HMA induction can be regarded as a *de facto* standard of care for all patients with *TP53*m AML outside of a clinical trial (DiNardo et al., 2019; DiNardo C. D. et al., 2020; Shallis et al., 2021). Compared to HMAs alone, inclusion of venetoclax consistently improves the response rates and short-term disease control in *TP53*m AML despite showing only modest effects on overall survival(OS). These gains may translate into clinically meaningful benefits, such as higher rates of cytoreduction, as a bridge to allogeneic hematopoietic stem cell transplantation and improved quality of life, thereby supporting venetoclax usage even in the absence of persistent survival benefits.

While venetoclax + HMA is arguably the best available therapy for *TP53*m AML, the clinical benefits are clearly short-lived and subpar. Addition of venetoclax to HMA improves the CR from 13% (HMA alone) to 33% (venetoclax + HMA) but does not translate to improved mOS, which is currently 6 months for both groups (same as IC) (Daver et al., 2023). A recent propensity matched analysis of earlier phase II/III trials reported an improved mOS rate with decitabine + cedazuridine than parenteral HMA (13.1 vs. 8.0 months), suggesting the rationale for preferring oral monotherapy over parenteral HMA ± venetoclax, although there was no comparison with a venetoclax-inclusive regimen (Urrutia et al., 2024). Overall, these findings question whether venetoclax-inclusive regimens truly represent a reference standard for *TP53*m AML (Pollyea et al., 2022).

Furthermore, these data ultimately suggest resistance (both primary and secondary) to venetoclax as a core feature of *TP53*m AML, similar to monocytic differentiated, *FLT3-*ITD mutated, and *RAS-*mutated AML, that needs to be better understood (Pei et al., 2020; Mali et al., 2020; Zhang et al., 2022; Diamantidis, 2025). Given the increasing use of venetoclax + HMA, there is potential for even greater use depending on the results of multiple trials randomizing patients with higher-risk disease to either IC or venetoclax + HMA (e.g., PARADIGM or NCT04801797 reported above; MM1YA-S01 or NCT05554406 recruiting for IC vs. venetoclax + HMA vs. IC + venetoclax); given the increasing recognition that *TP53*m MDS with increased blasts is a functional equivalent of AML, the biological underpinnings of venetoclax resistance in *TP53*m AML are nuanced yet critical for understanding (Khoury et al., 2022; Arber et al., 2022; Shallis et al., 2023a; Shallis et al., 2023b).

Extant reviews of *TP53*m AML have largely emphasized the defects in mitochondrial apoptotic priming and genomic instability as drivers of treatment resistance. In contrast, the present review highlights emerging evidence that resistance to venetoclax may also be related to post-mitochondrial caspase blockade. Furthermore, we discuss recent observations of *TP53* mutations within immune subpopulations, including T and NK cells, and their potential contributions to immune exhaustion. Finally, we integrate these insights with evolving clinical data to examine the rationale for venetoclax dosing and duration de-escalation strategies in *TP53*m AML to better inform future rational combinations and trial designs.

## 2 BCL-2 family and venetoclax

There are two main apoptotic pathways for each cell, namely, the intrinsic pathway (in response to cellular stress via alterations to the mitochondrial permeability) and extrinsic pathway (external signals like death receptor signaling) (Zimmermann and Green, 2001). The BCL-2 family comprises a group of structurally related proteins that regulate mitochondrial outer-membrane permeabilization (MOMP) as the commitment step of intrinsic apoptosis. The members of this family interact through conserved α-helical BCL-2 homology (BH) domains to either preserve the mitochondrial integrity or promote apoptotic pore formation. Based on the number and arrangement of these BH domains, the BCL-2 proteins are classified into distinct functional subgroups with proapoptotic or antiapoptotic roles (Czabotar and Garcia-Saez, 2023).

The BCL-2 family of proteins regulates the intrinsic pathway through an elegant interplay of proteins grouped according to functions and number of domains (conserved BH regions): multidomain antiapoptotic proteins (BCL-2, BCL-XL, BCL-W, MCL-1, BCL-2A1, and BCLB), multidomain proapoptotic proteins (BAK, BAX, and BOK), and single-domain proapoptotic proteins (BID, BIM, BAD, BIK, NOXA, PUMA, BMF, and HRK) (Vogler et al., 2025). The multidomain antiapoptotic proteins inhibit the multidomain proapoptotic proteins; furthermore, the single-domain (BH3-only) proapoptotic proteins can serve dual roles by either activating the multidomain proapoptotic proteins directly or acting as a neutralizing sink for the antiapoptotic proteins.

**FIGURE 1:**
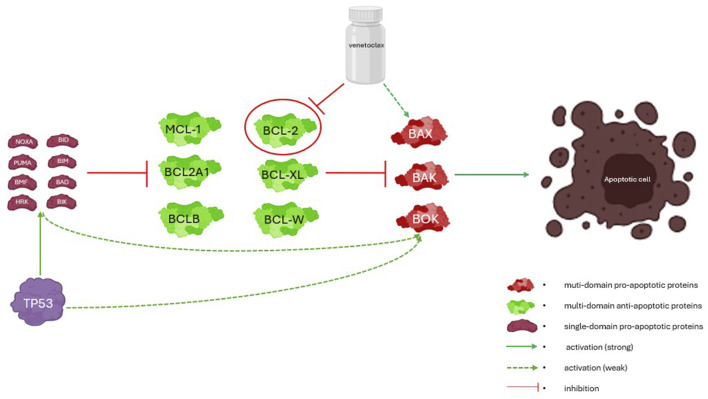
Simplified mechanistic scheme of the interplay among BCL-2, wild-type *TP53*, and venetoclax. The BCL-2 family of proteins regulates mitochondrial outer-membrane permeabilization (MOMP) through interactions mediated by conserved BCL-2 homology (BH) domains. The antiapoptotic members (e.g., BCL-2, BCL-XL, and MCL-1) sequester proapoptotic effectors (BAX and BAK) to preserve mitochondrial integrity. The BH3-only proteins either activate BAX/BAK directly or neutralize the antiapoptotic proteins to tip the balance toward apoptotic pore formation and cell death.

The prototype *BCL-2* gene product was recognized as an attractive drug target because of its overexpression in malignant cells and redundancy in normal cells, thereby creating a theoretical therapeutic window (Letai, 2008). However, early agents targeted the broader BCL-2 protein family rather than BCL-2 specifically. For example, navitoclax is a potent dual inhibitor of BCL-2 and BCL-XL; its clinical development was limited by on-target toxicity related to BCL-XL inhibition, most notably dose-limiting thrombocytopenia, owing to which it failed to achieve meaningful clinical success (Debrincat et al., 2015). The need for more selective agents that did not inhibit BCL-XL yet retained potency against BCL-2 led to the design and development of venetoclax (Cang et al., 2015). Venetoclax was reengineered from its predecessor navitoclax to create a highly selective BCL-2 inhibitor, thus avoiding concomitant BCL-XL inhibition and mitigating the prohibitive thrombocytopenia observed with navitoclax. Venetoclax works as a single-domain BH3 mimetic agent that attaches to the BH3 binding groove of BCL-2 and removes its inhibition of multidomain proapoptotic proteins (Souers et al., 2013). Similar to other BH3-only single-domain proapoptotic proteins, venetoclax can directly activate the executioner proteins; this interplay and related interactions are summarized in Figure 1.

## 3 Wild-type *TP53* and BCL-2 interaction

*TP53* primarily influences the intrinsic apoptotic pathway but has been implicated in the extrinsic pathway as well; p53 is activated by cellular stress and/or DNA damage, which prompt the former to transcriptionally induce single-domain proapoptotic proteins (e.g., PUMA and NOXA) that consequently act as neutralizing sinks for antiapoptotic proteins like BCL-2. This overwhelms the buffering capacity of BCL-2 and allows the multidomain proapoptotic proteins (BAK and BAX) to increase MOMP, thereby promoting the intrinsic pathway apoptosis cascade (Cory and Adams, 2002). Beyond the transcriptional activation of proapoptotic proteins, p53 also translocates to the mitochondria directly and promotes BAX/BAK activation (Chipuk et al., 2004). The combined downstream effects of p53 activation lower the apoptotic threshold of the cells. In wild-type *TP53* cells under oncogenic stress, venetoclax acts in conjunction with transcriptionally induced proapoptotic proteins (e.g., PUMA and NOXA) to overwhelm the BCL-2 buffering capacities of the multidomain proapoptotic proteins (BAK and BAX). Thus, by lowering the apoptotic threshold, the wild-type *TP53* cells are primed for venetoclax-induced cell death in the absence of rescue by other mitigating prosurvival pathways (Souers et al., 2013; Konopleva et al., 2016) (Figure 1).

## 4 Mechanisms of BCL-2 inhibitor resistance in *TP53*m AML

*TP53*m AML is strongly enriched for complex karyotypes (∼70% of patients), reflecting the underlying genomic instability that fuels clonal heterogeneity (Rücker et al., 2012). There are likely multiple subclones present at diagnosis with varied levels of sensitivity to venetoclax that ultimately facilitate rapid selection of preexisting resistant subclones under chemotherapeutic pressure (not unique to venetoclax). However, other mechanisms not inherent to the clonal topography have also been implicated. Increased expressions of non-BCL-2 antiapoptotic proteins, selection of alternate clones, increased genomic instability, and lineage plasticity are some of the mechanisms postulated to account for venetoclax resistance (Nwosu et al., 2024; Bo et al., 2017). Herein, we discuss these resistance mechanisms specifically in the *TP53*m AML subgroup.

### 4.1 Shifting from BCL-2 dependence and post-mitochondrial executioner phase disruption

*TP53*m AML is heterogeneous in its antiapoptotic protein dependency. While venetoclax selectively targets BCL-2, other antiapoptotic pathways like those driven by MCL-1 and BCL-XL may become dominant. Waclawiczek et al. (2023) proposed a “mediators of apoptosis combinatorial score” or MAC score that links the ratio of protein expressions of BCL-2, BCL-XL, and MCL-1 in leukemic stem cells to predict responses to venetoclax (BCL-2/BCL-XL + MCL-1). Low MAC scores suggesting increased dependence on BCL-XL and MCL-1 were linked to *TP53*m AML in a prospectively validated cohort (Renders et al., 2025). The MAC scores are dynamic and generally lower at relapse, suggesting further selection of MCL-1 and BCL-XL pathways at the expense of BCL-2; the cellular addiction of MCL-1 and BCL-XL pathways appears to mediate both primary and secondary resistances. Selection of non-BCL-2 apoptotic pathways is the most extensively studied mechanism of venetoclax resistance in *TP53*m AML, with multiple independent studies corroborating its predictive value (Vänttinen et al., 2023; Nechiporuk et al., 2019).

Mamdouh et al. (2025) suggest that compensatory upregulation of the proapoptotic BIM pathway preserves the ability for MOMP; however, the disruption in activation of post-mitochondrial executioner caspases is suggested as the functional step for venetoclax + HMA resistance. This is a significant observation as a prior work focused on defects in the mitochondrial phase of apoptosis rather than the executioner phase (Vänttinen et al., 2023).

### 4.2 Loss of TP53 function and impact on venetoclax susceptibility

AML cells rely on the balance between proapoptotic and antiapoptotic proteins for sustained survival. Under oncogenic stress, wild-type *TP53* transcriptionally induces single-domain (BH3 only) proapoptotic proteins (e.g., PUMA and NOXA) that primarily bind to the antiapoptotic proteins (e.g., BCL-2) and neutralize their inhibitive hold on the multidomain proapoptotic proteins (BAK and BAX) (Vousden and Lane, 2007). As a BH3-mimetic agent, venetoclax also acts in a similar manner. Thus, the combination of p53-induced transcriptional products and venetoclax shows at least an additive if not a synergistic effect in favor of apoptosis. This additive effect is not present in *TP53*m AML and is therefore likely responsible for primary venetoclax resistance. *TP53* mutations result in a protein product that either fails to localize to the mitochondria or interacts productively with BAX/BAK (Mihara et al., 2003).

In addition to loss of wild-type-*TP53*-induced transcription of proapoptotic proteins, *TP53*m AML appears to be subjected to a loss of p53-mediated direct activation of BAX/BAK. Direct translocation to the mitochondria and promotion of BAX/BAK activation are additional mechanisms by which p53 tips the cellular balance toward apoptosis (Chipuk et al., 2004). These mechanisms appear to be transcription-independent and further reduce the apoptotic thresholds of the cells in the presence of venetoclax.

### 4.3 Lineage plasticity and monocytic differentiation

Data from single-cell DNA and protein sequencing along with single-cell mass cytometry on longitudinal samples from patients with *TP53*m AML have been used to elucidate the resistance mechanisms. Ayoub et al. (2024) found that resistant clones shifted from CD34^+^ blasts to CD16^+^ monocytic cells and underwent monocytic differentiation thereof as an escape mechanism in response to venetoclax-based treatment; venetoclax therapy is known to be less effective against AML with monocytic differentiation, secondary to a distinct transcriptome, loss of BCL-2 expression, and increased reliance on MCL-1 (Ayoub et al., 2024; Pei et al., 2020).

### 4.4 Metabolic reprogramming

In addition to direct modulation of the apoptotic/epigenetic pathways, the venetoclax + HMA combination partly exerts its effect by suppressing oxidative phosphorylation (OXPHOS) in AML cells (Pollyea et al., 2018). However, metabolic plasticity allows the AML cells to adjust their energy pathways for adaptation and survival under drug pressure (Jones et al., 2018). In particular, *TP53*m AML cell lines have been shown to have enhanced OXPHOS activities and hence resistance to venetoclax-based therapy (Daver et al., 2022; Nechiporuk et al., 2019; Vadakekolathu et al., 2024).

The other metabolic compensations include increased fatty acid levels in the AML cell lines (Nechiporuk et al., 2019). Metabolic shifting toward fatty acid oxidation (FAO) as a compensation for inhibition of amino-acid metabolism by venetoclax + HMA is postulated to be another mechanism of venetoclax resistance in this molecular subgroup (Stevens et al., 2020).

### 4.5 Immunosuppressive and exhausted microenvironment

Disease control in AML treated with venetoclax is likely a function of drug-driven apoptosis and immune-microenvironment-driven surveillance/clearing of the residual blasts. However, *TP53*m AML is known to be associated with an immunosuppressive phenotype; this is a result of loss of negative regulators (e.g., the p53 transcription target *miR-34a*) of the proliferative gene products and increase in the number of immunosuppressive cells (e.g., Tregs) (Sallman et al., 2020). This may contribute to the clinically observed early response but eventual disease breakthrough, including under the therapeutic pressure exerted by venetoclax.

Furthermore, emerging data from *TP53*m AML indicate the presence of *TP53* mutations in lymphoid subpopulations, namely, T and NK cells identical to those found in leukemic blasts from the same patients (Ayoub et al., 2024; Li et al., 2024). *TP53*m-T/NK cells have been associated with enhanced proliferative and activation profiles but impaired functionality leading to exhaustion (Li et al., 2024). While this does not exert a direct effect on the venetoclax-driven intrinsic apoptotic pathway, the immune escape likely explains the early but short-lived responses observed with frontline HMA + venetoclax in *TP53*m AML.

### 4.6 Acquired mutations

Until recently, *BCL-2* mutation as a venetoclax resistance mechanism was largely described in the context of CLL-directed therapy (Blombery et al., 2025). A more recent work by Brown et al. (2025) reported acquired *BCL-2* variants (e.g., D103E, F104L, and V148L) that emerged secondary to venetoclax exposure and reduced its affinity to BCL-2. However, the prevalence of this phenomenon in AML is unclear at this point, and authors have postulated that this is an underreported mechanism of treatment failure.

Moujalled et al. (2023) reported detection of mutations in the apoptotic effector *BAX* in approximately 17% of patients with AML who relapsed while on venetoclax-inclusive therapy; among these patients, 29% of the population had *TP53*m AML. The mutations included frameshift/non-sense variants (R34*, W139*, W188*, E41Gfs*33, and A117Gfs*22) leading to truncated proteins or messenger RNA decay as well as missense mutations (G23R, G166E, P168A, and W170S) predicted to impair *BAX* functioning via its retention in the cytosol. *BAX* gene inactivation (along with *TP53* inactivation) has been previously identified as a key mediator of venetoclax resistance in AML through genome-wide CRISPR/Cas9 screening in AML cell lines (Nechiporuk et al., 2019).

## 5 Therapeutic opportunities to mitigate venetoclax resistance

The outcomes for patients with *TP53*m AML are poor (mOS of 6–7 months) and have been shown to be independent of age, fitness (or “eligibility” for IC), and type of treatment administered (i.e., IC or venetoclax + HMA); these have largely favored the standard-of-care regimen associated with the least toxicity or HMA ± venetoclax (Sallman and Stahl, 2025; Daver et al., 2023). There is a dire need to improve the therapeutic options for this patient population through incorporating efficient novel therapies as well as overcoming venetoclax resistance. This will likely require moving beyond BCL-2-centric approaches to address the broader survival circuitry of the disease. Some of the potential strategies include targeting alternative antiapoptotic dependencies and differentiation states that emerge under venetoclax pressure as well as disrupting the immune-evasive microenvironment characteristic of *TP53*m AML. Herein, we discuss some of these possibilities and summarize them in Table 1.

**TABLE 1:** Mechanisms of venetoclax resistance in *TP53-*mutated (*TP53*m) acute myeloid leukemia (AML).

Number	Category	Mechanistic rationale	Comment
1	Shift away from BCL-2 dependence	Apoptotic escape mediated by redundant antiapoptotic proteins like MCL-1 (Waclawiczek et al., 2023)	Direct MCL-1 inhibitors (e.g., MIK665) and indirect MCL-1 inhibitors (e.g., deubiquitinase, CDK9, and SPHK1 inhibitors) are under investigation
2	Acquired mutations of the *BCL-2* gene	*BCL-2* variants (e.g., D103E, F104L, and V148L) that emerge secondary to venetoclax exposure and reduce its affinity for BCL-2 (Brown et al., 2025)	Unclear prevalence, increased targeted testing should be considered; could be a biomarker of venetoclax futility
3	Post-mitochondrial executioner phase disruption	Decoupling of the mitochondrial and executioner phases of apoptosis (Mamdouh et al., 2025)	Need to investigate the critical step for this impaired executioner caspase activity
4	*BAX* gene inactivation	Apoptotic effector gene inactivation (Moujalled et al., 2023)	Unclear prevalence, increased targeted testing should be considered; could be a biomarker of venetoclax resistance
5	Loss of wild-type-*TP53-*induced transcription of proapoptotic proteins	Decreased levels of proapoptotic proteins (e.g., PUMA and NOXA) and resulting increased apoptotic threshold for venetoclax (Mihara et al., 2003)	Possible role of MDM2 inhibitors in monoallelic *TP53* loss
6	Loss of p53-mediated direct activation of BAX/BAK	*TP53*m results in a protein product that either fails to localize to the mitochondria or interact productively with BAX/BAK and the resulting increased apoptotic threshold for venetoclax (Chipuk et al., 2004)	Possible role of MDM2 inhibitors in monoallelic *TP53* loss
7	Lineage plasticity and monocytic differentiation	Resistant clones shift from CD34^+^ blasts to CD16^+^ monocytic cells (Ayoub et al., 2024)	Differences could be leveraged in the future as differential targets for antibodies, antibody–drug conjugates, or CAR-Ts. Alternately, sequential evaluation to explore monocytic differentiation could be used to change treatment strategy as it signifies venetoclax resistance
8	High genomic instability and clonal heterogeneity	Strongly enriched for complex karyotypes (∼70% of patients). Multiple subclones are likely present at diagnosis with varied levels of sensitivity to venetoclax, facilitating rapid selection of preexisting resistant subclones (Nwosu et al., 2024)	*Ex vivo* venetoclax sensitivity profiling could help gauge expected responses on a patient-specific basis
9	Enhanced oxidative phosphorylation (OXPHOS)	Venetoclax partly exerts its effect by suppressing OXPHOS. *TP53*m AML cell lines show resistance to this phenomenon partly by increased fatty acid oxidation (FAO) (Stevens et al., 2020)	Inhibition of FAO is being studied as means to resensitize cells to venetoclax
10	Immunosuppressive and exhausted microenvironment	*TP53*m-T/NK cells are associated with enhanced proliferative and activation profiles but impaired functionality leading to exhaustion (Sallman et al., 2020)	Near-term possible utility as biomarkers to justify earlier transition to trial-based immune combinations or cellular strategies

### 5.1 Optimizing venetoclax exposure

Given the debate on whether venetoclax should be offered to patients with *TP53*m AML and the profound biological heterogeneity observed in the molecular subset, *ex vivo* venetoclax sensitivity profiling may a pragmatic approach to gauging the expected responses on a patient-specific basis (Kuusanmäki et al., 2019). This approach is particularly appealing for cytoreduction or bridging strategies to control the disease while awaiting transplantation or enrollment in definitive trials.

Among patients committed to receiving venetoclax, the optimal dosing schedule that maximizes efficacy, reduces toxicity, and prevents emergence of resistant clones is still under debate. Considering that there is no significant OS benefit associated with the addition of venetoclax to HMA despite the significantly better ORR and DoR, it is likely that venetoclax-related myelosuppression and the resulting infections/bleeding contribute at least partly to the decreased OS (Daver et al., 2023). There is demonstrated evidence of an increase in the size of the *TP53*m clone and emergent biallelic *TP53* defects at the time of relapse in response to venetoclax therapeutic pressure (DiNardo C. et al., 2020). Additionally, Gruszczynska et al. (2024) examined the rate of founding clone clearance in *TP53*m AML and found no benefits to the decitabine + venetoclax combination over decitabine alone. Consequent to these findings, some AML experts advocate omitting venetoclax, especially in the case of patients who are not intended for allogeneic hematopoietic stem cell transplantation (alloHSCT); the countervailing opinion is that the time spent in CR, which can be improved by venetoclax combination therapy, may be associated with better freedom from transfusion dependence, reduced infection/bleeding, and improved health-related quality of life.

Together, these data strongly support the need to explore reductions to venetoclax exposure both pre- and post-remission. Multiple retrospective studies have evaluated abbreviated pre-remission venetoclax schedules (21, 14, or 7 days per cycle) and demonstrated similar remission rates and OS to VIALE-A dosing, including in *TP53*m AML (Karrar et al., 2024; Willekens et al., 2025; Aiba et al., 2023). Similarly, post-remission venetoclax limited to 7 days per cycle has been accepted in other investigational triplets (e.g., HMA + venetoclax + gilteritinib; NCT06317649). Therefore, venetoclax de-escalation particularly in minimal residual disease (MRD)-negative *TP53*m AML warrants further study, given the cumulative toxicity and uncertain long-term benefit. At this point, venetoclax omission in *TP53*m AML remains investigational.

### 5.2 Targeting non-BCL-2 survival pathways

As described previously, alternative pathways like MCL-1 are implicated in the escape from apoptosis. Multiple agents are currently in various phases of preclinical development or trials to address this pathway redundancy. However, many of these efforts have succumbed to drug development hurdles. Among the direct MCL-1 inhibitors, the MIK665 phase I/II trial was terminated for strategic reasons. Murizatoclax and AZD5991 development were suspended owing to cardiotoxicity that raised concerns that MCL-1 inhibition and related cardiotoxicity maybe a class effect (Nwosu et al., 2024). Zhang et al. (2022) showed that the RAS/MAPK pathway leads to increased stability and levels of MCL-1. Hence, inhibition of the RAS/MAPK pathway could be an indirect method of decreasing MCL-1 level while potentially mitigating the cardiotoxicity concerns. Inhibition of deubiquitinase, CDK9, and SPHK1 are some of the other postulated indirect approaches for inhibiting MCL-1 and may soon be studied as combination therapies in *TP53*m AML (Nwosu et al., 2024).

### 5.3 FAO inhibition

Pharmacologic FAO inhibition has been shown to restore venetoclax sensitivity in resistant AML, which is subject to a metabolic rewiring toward FAO that ultimately circumvents the amino-acid dependency exploited by venetoclax + HMA. Targeting FAO by agents like etomoxir in combination with venetoclax serves as a rational therapeutic strategy for future study (Stevens et al., 2020).

### 5.4 Addressing lineage plasticity and monocytic differentiation

Owing to the single-cell findings that *TP53*m resistant clones shift from CD34^+^ blasts to CD16^+^ monocytic cells, the dynamic topographies of cell surface molecules offer opportunities for incorporating targeted antibody–drug conjugates or chimeric antigen receptor products in future studies (Ayoub et al., 2024). Alternately, sequential evaluation to explore monocytic differentiation could be used to change the treatment strategy as it signifies venetoclax resistance.

### 5.5 *In vitro* sensitivity-guided therapy and bridge strategies

Given the profound heterogeneity of *TP53*m AML, *ex vivo* venetoclax sensitivity profiling is a pragmatic approach to gauging expected responses on a patient-specific basis (Kuusanmäki et al., 2019). This approach is particularly appealing for cytoreduction or bridging strategies to control the disease while awaiting transplant or enrollment into definitive trials.

### 5.6 Novel therapies to surmount venetoclax resistance mechanisms

Many therapeutic approaches unrelated to or circumnavigating the hypothesized mechanisms of venetoclax resistance in *TP53*m AML have been proffered and/or tested, such as classical and non-classical immune checkpoint inhibition (e.g., anti-PD1/PDL1, anti-CD47) and mutant p53 “refolding” agents (e.g., APR-246/eprenetapopt), but very few of these have attempted to directly abrogate the resistance (Zawacka, 2024).

Agents leveraging action mechanisms that bypass classical chemoresistance pathways, including those utilized by venetoclax, are being prioritized. Lintuzumab-Ac225 is an alpha-particle-emitting radionuclide (Actinium-225 or Ac225) conjugated to the humanized monoclonal antibody lintuzumab, where the latter targets CD33 that is highly expressed on malignant tissues across an array of myeloid malignancies. The high-energy and short-path-length α-particle delivery to tumor cells promoted by this agent leads to double-strand DNA breaks, generation of reactive oxygen species, and antibody-dependent cellular cytotoxicity (ADCC)/phagocytosis, consequently leading to leukemic cell death (Garg et al., 2021; Sutherland et al., 2009). A potent and dose-dependent anti-leukemic effect was shown across several AML cell lines irrespective of the mutational subgroup, including *TP53*m cell lines (i.e., Kasumi-1 and HL-60) (Chin et al., 2025). Synergy with HMA as well as venetoclax has been demonstrated, where the latter may be related to the ability of lintuzumab-Ac225 to deplete the antiapoptotic protein MCL-1 (Zerp et al., 2009; Hara et al., 2005). Among 12 trial patients with the *TP53*m disease in combination with CLAG-M (cladribine, cytarabine, G-CSF, and mitoxantrone) in the relapsed/refractory (R/R) AML setting, 50% achieved CR/CRi with 75% of responders achieving MRD negativity and a mOS of 10 months; this indirectly exceeds the historical estimate among patients in the frontline setting, where the responses and DoR (to approximate OS) are classically superior (Abedin et al., 2025). These data appear to support the agent or action mechanism as being *TP53* agnostic. Accordingly, the combination of lintuzumab-Ac225 with ASTX727 (decitabine + cedazuridine) + venetoclax is being studied in a National Cancer Institute (NCI)-sponsored phase 1 trial that is actively recruiting patients with newly diagnosed AML who are otherwise “unfit” for intensive therapy without biomarker restriction (i.e., *TP53* mutation) (NCI 10653, NCT06802523).

Arsenic trioxide is another agent of interest that is hypothesized to promote structural rescue of certain p53 variants, thereby sensitizing *TP53*m AML cells to chemotherapy agents (Chen et al., 2021). At least one clinical trial (NCT06778187) is currently evaluating arsenic trioxide in combination with venetoclax. Mutant p53 degraders like HSP90 inhibitors (e.g., ganetespib) and HDAC inhibitors (e.g., vorinostat) promote destabilization and degradation of oncogenic mutant p53, which is one of the strategies to eliminate mutant p53 gain-of-function forms (Moore et al., 2025). Atorvastatin has been shown to promote degradation of misfolded mutant p53 proteins and is being evaluated in another trial (NCT03560882). Other agents like MDM2 inhibitors, including idasanutlin, navtemadlin, milademetan, and APG-115, may also play roles in monoallelic *TP53* loss by potentiating the function of the wild-type copy (Zawacka, 2024).

## 6 Conclusion

Although venetoclax-based combination therapies have been shown to improve response rates compared to HMA monotherapies and achieve survival comparable to IC, they confer very little OS benefit in patients with *TP53*m AML. This limitation reflects both treatment-related myelosuppression with attendant clinical complications as well as the fundamental intrinsic chemoresistance and venetoclax resistance driven by *TP53* mutations. Although venetoclax de-escalation or omission strategies are under investigation in this high-risk population, there is a critical need for novel therapies capable of overcoming the venetoclax resistance mechanisms.
